# Detection of Longer Leukocyte Telomere Length in Patients With Bone Sarcomas

**DOI:** 10.14740/wjon2684

**Published:** 2026-05-08

**Authors:** Mariana Chantre-Justino, Rafaele Tavares Silvestre, Lucas Delmonico, Gilda Alves, Maria Helena Faria Ornellas, Caroline Rotilho, Amanda Cavalcanti, Jamila Alessandra Perini, Nina Carrossini Bastos, Eliane Luz, Rafael Pinheiro, Bruna Canteri Delocco, Anabela Cunha Caruso, Ana Cristina de Sa Lopes, Walter Meohas

**Affiliations:** a Research Division, National Institute of Traumatology and Orthopaedics, Rio de Janeiro 20940-070, Brazil; b Circulating Biomarkers Laboratory, Pathology Department, Faculty of Medical Sciences, Rio de Janeiro State University, Rio de Janeiro 20550-170, Brazil; c Cytogenetic Laboratory, Cell and Gene Therapy Program, Instituto Nacional de Cancer (INCA), Rio de Janeiro 20230-130, Brazil; d Research Laboratory of Pharmaceutical Sciences (LAPESF), Rio de Janeiro State University, Rio de Janeiro 23070-200, Brazil; e Divisao de Patologia, Laboratorio de Patologia Molecular, National Cancer Institute (INCA), Rio de Janeiro 20220-400, Brazil; f Specialized Care Center for Orthopedic Oncology, National Institute of Traumatology and Orthopaedics, Rio de Janeiro 20940-070, Brazil

**Keywords:** Telomere length, Leukocyte telomere length, Bone sarcomas

## Abstract

**Background:**

Bone sarcomas are rare and heterogeneous malignant neoplasms of mesenchymal origin, often associated with poor clinical outcomes. Being rare neoplasms, a comprehensive analysis of the molecular mechanisms involved in the tumor biology of bone sarcomas is still lacking. Telomeres are repetitive nucleotide sequences (TTAGGG)n at chromosome ends playing an essential role in genome stability, and their dysfunction has been associated with several human diseases, including cancer. This study aimed to assess telomere dynamics in pediatric and adult patients with bone sarcomas.

**Methods:**

The measurements of relative telomere length (RTL) in peripheral blood leukocytes were evaluated by quantitative polymerase chain reaction (qPCR) in 44 patients with newly diagnosed, histologically confirmed, treatment-naive bone sarcomas. The control group comprised 50 cancer-free individuals.

**Results:**

Overall, we observed significantly longer RTL in patients compared to controls (P = 0.02). RTL was also significantly longer in male patients compared to male controls (P = 0.01). Among patients, no significant association was observed between RTL and age group (pediatric vs. adult), sex (male vs. female), and outcome events (recurrence, metastasis or death).

**Conclusions:**

Our findings indicate that longer leukocyte telomere length in patients, compared with cancer-free controls, may be associated with increased susceptibility to bone sarcoma.

## Introduction

Bone sarcomas constitute a heterogeneous, rare, and clinically complex group of malignant neoplasms of mesenchymal origin accounting for about 1% of human cancers worldwide [1–4]. Osteosarcoma, Ewing sarcoma, and chondrosarcoma are the most prevalent malignant bone tumors [1, 2, 5]. These rare neoplasms affecting children and adults may arise in different anatomical sites and display variable biological behavior. Patients with metastatic disease often experience poor clinical outcomes, making management particularly challenging. Therefore, a comprehensive analysis of the molecular biology underlying bone sarcomas remains incomplete.

Telomeres are repetitive nucleotide sequences (TTAGGG)n at the ends of chromosomes associated with a protein complex. Telomeres play an essential role in genome stability by preventing chromosomal end-to-end fusion and misrecognition of the ends as sites of DNA strand breaks [6]. Progressive telomere length (TL) shortening occurs with biological aging in normal somatic cells up to reach critical TL, which leads to replicative senescence [7]. However, most cancer cells can evade this process by maintaining TL and therefore continue to divide with extensive accumulation of mutations. Telomere dysfunction has been associated with increased cancer risk and worse prognosis, mediated by accelerated telomere shortening [8, 9] or longer TL [10, 11], creating a TL paradox in cancer [12]. TL maintenance can occur through two distinct mechanisms: 1) telomerase activity via human telomerase reverse transcriptase (hTERT) reactivation; and 2) alternative lengthening of telomeres (ALT). Telomerase activation does not appear to be the predominant pathway for TL maintenance in sarcoma, as the ALT mechanism is commonly observed in these tumors of mesenchymal origin [13–18].

Due to the biological complexity and clinical heterogeneity of these tumors, a comprehensive analysis of the TL dynamics in tumorigenesis and its clinical relevance in sarcomas is still lacking. Leukocyte telomere length (LTL) is widely measured in population-based studies due to the accessibility of peripheral blood cells and is used as a surrogate marker of systemic telomere regulation in relation to cancer risk [19, 20]. In this study, we aimed to evaluate the relative telomere length (RTL) in peripheral blood lymphocytes by quantitative polymerase chain reaction (qPCR) in pediatric and adult patients with bone sarcomas.

## Materials and Methods

### Study participants

This study recruited 44 patients with newly diagnosed, histologically confirmed, treatment-naive bone sarcomas between October 2022 and August 2024 at the Specialized Care Center for Orthopedic Oncology, National Institute of Traumatology and Orthopedics, Rio de Janeiro, Brazil. Demographic and clinical information were collected from each patient, as follows: age at diagnosis, sex, histological subtype of sarcoma, primary site of tumor, and outcome events (recurrence, metastasis or death). The control group comprised 50 cancer-free individuals. This study was approved by the Ethics and Research Committee of the Institution (CAAE: 60632822.4.0000.5273) and was conducted in compliance with the ethical standards of the responsible institution on human subjects as well as with the Helsinki Declaration. The written informed consent was obtained from each healthy donor, adult patient, and legal guardian of pediatric patients (< 18 years old).

### Sample collection and DNA isolation

Peripheral blood samples from all participants were collected in ethylenediaminetetraacetic acid (EDTA) tubes. Genomic DNA was extracted from the buffy coat of blood using Quick-DNA Miniprep Kit (D3025, Zymo Research Corp.), according to the manufacturer’s protocol.

Genomic DNA from fresh-frozen tumor tissue was extracted using the Quick-DNA Miniprep Kit (Zymo Research Corp., USA), whereas genomic DNA from formalin-fixed, paraffin-embedded (FFPE) tissue was extracted using the AllPrep DNA/RNA FFPE Kit (Qiagen, Germany), according to the manufacturer’s instructions.

After isolation, DNA was stored at –20 °C until further analysis. DNA was quantified in the spectrophotometer NanoDrop™ 2000c (Thermo Fisher Scientific Inc.).

### RTL

LTL was assessed by qPCR and expressed as RTL, calculated as the telomere to single copy gene (T/S) ratio, as previously described [21, 22]. The conversion from T/S ratio to base pairs was also calculated [23]. The qPCR reactions were carried out in duplicate and performed in a final volume of 25 µL using DNA sample (50 ng), primers for telomere (TEL) and human beta-globin (HBG, used as a single-copy reference gene for normalization), and Power SYBR™ Green PCR Master Mix (Life Technologies, Carlsbad, CA) in platform QuantStudio™ 3 Real-Time PCR System (Life Technologies, Carlsbad, CA). The thermocycling conditions and the primers sequences for TEL and HBG used in qPCR reactions were determined as previously described [22].

### Immunohistochemistry (IHC)

IHC was performed on 4-µm sections of FFPE specimens. The sections were incubated at 60 °C for at least 2 h, deparaffinized in xylene, and rehydrated through a graded series of ethanol. For antigen retrieval, slides were treated with Trilogy™ solution (Cell Marque, Rocklin, CA, USA) in a steamer for 30 min. After cooling to room temperature, the slides were processed using a commercial peroxidase polymer-based kit (Novolink Max Polymer Detection System, Leica Biosystems) according to the manufacturer’s instructions. Sections were incubated overnight at 4 °C with either anti-telomerase reverse transcriptase (TERT) (Clone 2C4, Invitrogen; 1:15,000 dilution) or anti-alpha thalassemia/mental retardation syndrome X-linked (ATRX) (polyclonal, Sigma; 1:800 dilution) primary antibodies. Harris hematoxylin was used for counterstaining.

All slides were evaluated by an experienced pathologist (ACC) using optical microscopy. Colon adenocarcinoma and adrenocortical carcinoma samples were used as positive controls for TERT and ATRX immunostaining, respectively. TERT and ATRX immunoexpression were evaluated according to staining pattern (diffuse, multifocal, or focal), intensity (strong, moderate, or weak), and subcellular localization (N: nuclear; C: cytoplasmic; Nu: nucleolar). ATRX loss was defined as the complete absence of nuclear staining in tumor cells in the presence of positive internal controls.

### Statistical analysis

The χ^2^ and Fisher’s exact tests were employed to evaluate the statistical significance of the relationships between RLT, demographic, and clinical parameters in patients and controls. To determine the significance of differences in RLT among evaluated groups, the *t*-test, Mann–Whitney test, and Kruskal–Wallis test were used. Data normality was assessed using the Shapiro–Wilk test. Linear regression analysis was applied to assess the correlation between age and RLT within these groups. Continuous variables were presented as the mean ± standard deviation (SD) if they were normally distributed, or as the median and range (minimum and maximum) if they were not normally distributed. All statistical analyses were conducted using GraphPad Prism 10.4.0. Associations with a P value less than 0.05 were deemed statistically significant.

## Results

### Characteristics of the participants

This study included 94 participants, 44 patients with bone sarcomas and 50 cancer-free subjects as control group. Bone sarcoma samples were represented by five subtypes: osteosarcoma (n = 27), chondrosarcoma (n = 7), Ewing sarcoma (n = 7), high-grade bone sarcoma (n = 2), and undifferentiated high-grade pleomorphic bone sarcoma (n = 1).

The patient cohort consisted of 27 adults (mean age: 44.81 ± 15.69 years; range: 20 to 75) and 17 children (mean age: 13.47 ± 3.50 years; range: 06 to 19), whereas the control group included 43 adults (mean age: 36.98 ± 12.53 years; range: 20 to 63) and seven children (mean age: 16.00 ± 1.63) years; range: 14 to 19). Osteosarcoma represented the most prevalent pediatric tumor. In total, six patients underwent limb amputation. Follow-up was lost in nine patients (20.5%), who were predominantly male (66.7%), had a mean age of 27.4 years (range: 13–59), and were mostly diagnosed with osteosarcoma (n = 8; one Ewing sarcoma); all were lost after biopsy. Among the remaining patients, 62.8% (22/35) experienced an outcome event (recurrence, metastasis or death) during the follow-up period, with the lung representing the site of all metastases. These clinical and demographic characteristics are summarized in Table 1. Primary tumor location was heterogeneous across the cohort, with the femur representing the most frequent site (47.7%), followed by the humerus (18.2%), as shown in Table 2.

**Table 1:** Clinical and Sociodemographic Characteristics of the Participants in This Study

Characteristics	Patients (n = 44)	Controls (n = 50)
Age at diagnosis, years, mean ± SD		
Overall	32.70 ± 19.79	34.04 ± 13.75
Adults	44.81 ± 15.69	36.98 ± 12.53
Children	13.47 ± 3.50	16.00 ± 1.63
		
Sex		
Female	17	22
Male	27	28
		
Tumor grade		
Low	4	NA
High	40	NA
		
Amputation of the limb		
Yes	6	NA
No	38	NA
		
Outcome at the last follow-up*		
Yes	22	NA
Recurrence	2	NA
Metastasis	4	NA
Recurrence + metastasis	3	NA
Death	7	NA
Metastasis + death	3	NA
Recurrence + death	1	
Recurrence + metastasis + death	2	
No	13	NA

*A total of 35 patients were evaluated during follow-up. SD: standard deviation; NA: not applicable.

**Table 2:** Primary Tumor Site of Bone Sarcomas in This Study

Primary site of tumor	Cases, n (%)
Femur	21 (47.7)
Humerus	8 (18.2)
Tibia	6 (13.6)
Fibula	4 (9.1)
Pelvis	2 (4.5)
Iliac	2 (4.5)
Scapula	1 (2.3)
Total	44 (100)

### RTL of the participants

**Figure 1:**
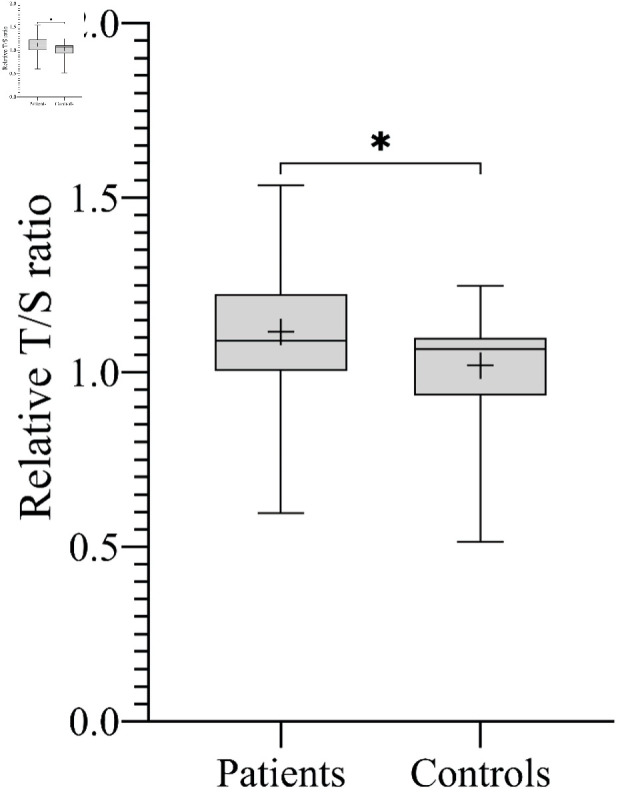
The mean RTL expressed by T/S ratio across all study participants. Boxplot showing that patients with sarcoma had significantly longer RTL compared to controls (*P = 0.02). T/S ratio of patients: 1.12 ± 0.20, corresponding to 5,967 ± 493 bp. T/S ratio of controls: 1.02 ± 0.14, corresponding to 5,735 ± 330 bp. bp: base pairs; RTL: relative telomere length; T/S ratio: telomere to single copy gene ratio.

**Figure 2:**
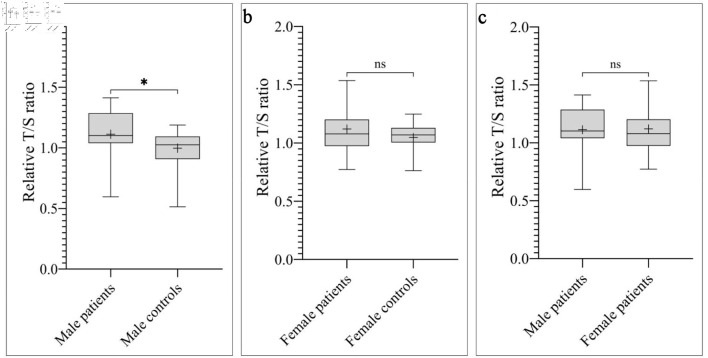
The mean RTL expressed by T/S ratio across gender. (a) Boxplot showing that male patients had significantly longer RTL compared to male controls (*P = 0.01). T/S ratio of male patients: 1.11 ± 0.19, corresponding to 5,960 ± 466 bp. T/S ratio of male controls: 1.00 ± 0.14, corresponding to 5,682 ± 346 bp. (b) No significant difference in RTL was observed between female patients and female controls (P = 0.57). T/S ratio of female patients: 1.12 ± 0.23, corresponding to 5,978 ± 548 bp. T/S ratio of female controls: 1.05 ± 0.13, corresponding to 5,802 ± 302 bp. (c) No significant difference in RTL was observed between male patients and female patients (P = 0.76). bp: base pairs; ns: not significant; RTL: relative telomere length; T/S ratio: telomere to single copy gene ratio.

Overall, the mean RTL was significantly longer in patients compared to controls (1.12 ± 0.20 vs. 1.02 ± 0.14, respectively; P = 0.02) (Fig. 1). Stratified analyses by histological subtype showed variable results across groups, with a statistically significant difference observed only in the Ewing sarcoma subgroup compared to controls (P = 0.0078). Regarding gender, the mean RTL was significantly longer in male patients compared to male controls (1.11 ± 0.19 vs. 1.00 ± 0.14, respectively; P = 0.01) (Fig. 2a). However, no significant difference in RTL was observed between female patients and female controls or between female patients and male patients (Fig. 2b, c).

**Figure 3:**
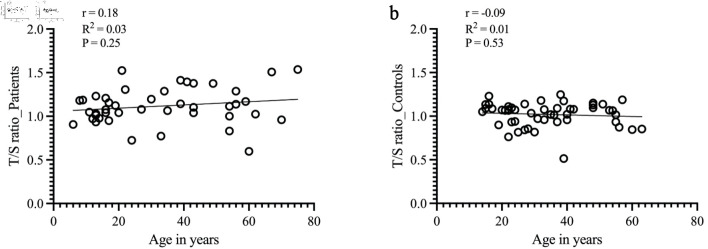
Correlation between the mean RTL and aging in patients and controls. Mean RTL was calculated by linear regression equation and is indicated by solid lines (y-axis = T/S ratio; x-axis = age in years). (a) For patients with sarcoma, no correlation was found between TL shortening and aging (Pearson correlation, r = 0.18, R^2^ = 0.03, P = 0.25). (b) For the control group, RTL was negatively correlated with age (Pearson correlation, r = -0.09, R^2^ = 0.01). However, no statistical significance was found (P = 0.5). RTL: relative telomere length; T/S ratio: telomere to single copy gene ratio.

Due to age-related TL progressive shortening, we next evaluated the relationship between RTL and age of the participants. No correlation was found between TL shortening and aging in the patient group (Fig. 3a). In the control group, a trend toward age-related TL shortening was observed, although it did not reach statistical significance (Fig. 3b).

### Association between RTL and clinical parameters

**Figure 4:**
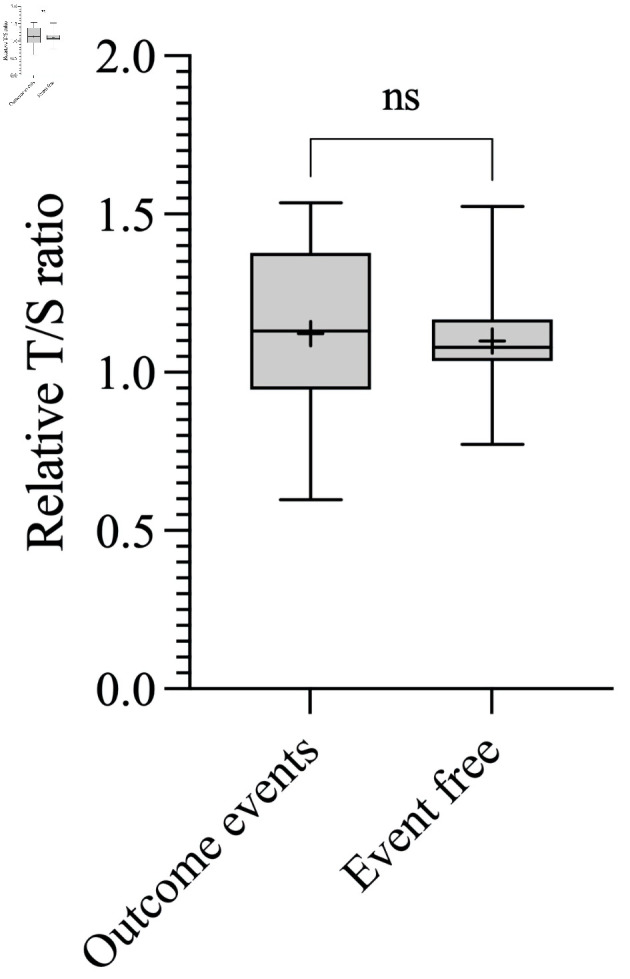
Associations between RTL and outcome events. Boxplot showing the T/S ratio of patients who experienced outcome event (1.12 ± 0.26, corresponding to 5,981 ± 616 bp) and T/S ratio of patients event free (1.10 ± 0.17, corresponding to 5,926 ± 407 bp). bp: base pairs; ns: not significant; RTL: relative telomere length; T/S ratio: telomere to single copy gene ratio.

We further assessed the association between the mean RTL and outcome events (recurrence, metastasis, or death), including only patients with available follow-up data. Nine patients without follow-up information were excluded from this analysis. No significant difference was found in the mean RTL between patients who experienced an outcome event and those who remained event-free (P = 0.91) (Fig. 4).

### TL in paired samples and TERT/ATRX IHC

To further explore telomere biology at the tumor level, a subset of patients with available tissue samples was analyzed. Paired assessment of TL (blood and tumor tissue) was available in 13 cases. Overall, T/S ratios in tumor tissue and peripheral blood showed a consistent pattern within individual patients, with only minor variations, suggesting a systemic component to TL regulation. These findings are summarized in Table 3.

**Table 3:** Telomere Length (T/S Ratio) in Peripheral Blood and Tumor Tissue, and Immunohistochemical Expression of TERT and ATRX

Patient	Sarcoma type	T/S blood	T/S tumor	TERT	ATRX
1	CS	0.96	1.03	NA	NA
2	CS	1.29	1.55	NA	NA
3	CS	1.11	1.02	Diffuse–strong–N/C	Diffuse–strong–N/C
4	CS	1.14	NA	Diffuse–strong–N	Diffuse–strong–N
5	OS	1.23	1.00	Multifocal–strong–N/Nu	Multifocal–strong–N
6	OS	1.19	1.11	Diffuse–strong–N	Focal–moderate
7	OS	1.39	1.19	NA	NA
8	OS	1.52	1.26	Diffuse–strong–N	Diffuse–strong–N
9	OS	1.04	1.06	NA	NA
10	OS	1.04	1.07	NA	NA
11	OS	1.38	1.09	Focal–C	Focal–weak–N
12	OS	0.95	1.22	Diffuse–strong–C	Diffuse–strong–N
13	ES	1.18	NA	Negative	Loss
14	ES	1.16	NA	Diffuse–moderate/strong–N/Nu	Diffuse–moderate/strong–N/Nu
15	ES	1.38	1.06	Multifocal–moderate–N	Diffuse–strong–N
16	ES	1.41	1.21	NA	NA

TERT and ATRX expression are reported as pattern (diffuse, multifocal, focal), intensity (strong, moderate, weak), and localization (N: nuclear; C: cytoplasmic; Nu: nucleolar). ATRX loss was defined as complete absence of nuclear staining in tumor cells in the presence of positive internal controls. NA: not available; CS: chondrosarcoma; OS: osteosarcoma; ES: Ewing sarcoma; T/S ratio: telomere to single copy gene ratio; TERT: telomerase reverse transcriptase; ATRX: alpha thalassemia/mental retardation syndrome X-linked.

**Figure 5:**
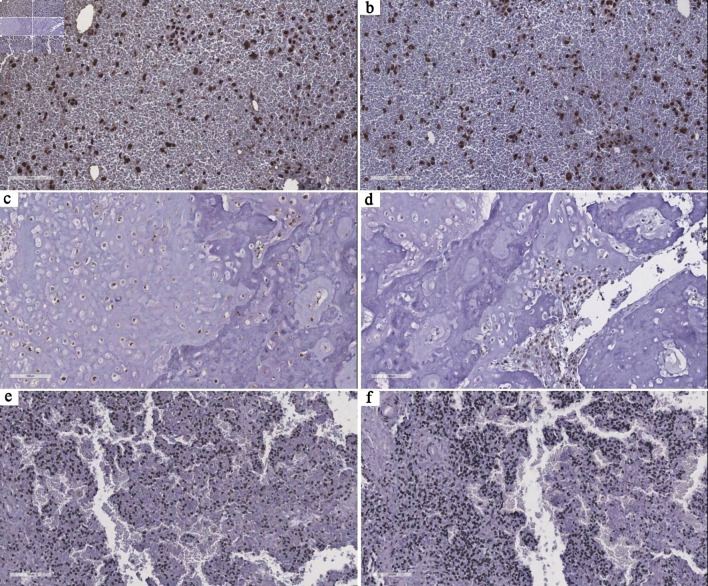
Representative immunohistochemical patterns of TERT and ATRX expression in tumor samples. (a, b) Case 8 shows diffuse, strong nuclear staining for both TERT and ATRX, consistent with a telomerase-associated profile. (c, d) Case 6 demonstrates diffuse and strong nuclear TERT expression with focal, moderate ATRX staining, highlighting intratumoral heterogeneity in telomere maintenance–related pathways. (e, f) Case 13 shows absence of TERT expression and complete loss of ATRX expression, suggesting a potential ALT phenotype. All images are at × 20 magnification. TERT: telomerase reverse transcriptase; ATRX: alpha thalassemia/mental retardation syndrome X-linked; ALT: alternative lengthening of telomeres.

To further characterize telomere maintenance mechanisms at the tumor level, immunohistochemical analysis of TERT and ATRX expression was performed in a subset of cases (n = 10). TERT expression predominantly exhibited a diffuse and strong staining pattern, mainly with nuclear localization, frequently accompanied by cytoplasmic and/or nucleolar staining. Multifocal and focal patterns were also observed, indicating variability in TERT expression across the cohort. ATRX expression was predominantly diffuse, strong, and nuclear, although some cases showed focal and weaker staining patterns. Notably, one case demonstrated absence of TERT expression and loss of ATRX, suggesting an ALT phenotype. These findings are shown in Figure 5 and summarized in Table 3.

## Discussion

Telomere biology in cancer is increasingly recognized as paradoxical, as both telomere shortening and aberrantly long telomeres have been associated with tumorigenesis. While accelerated telomere shortening may promote chromosomal instability in early stages, sustained telomere maintenance—through telomerase reactivation or ALT—enables malignant cells to bypass replicative senescence and achieve unlimited proliferative capacity [24]. In sarcomas, ALT appears to be more prevalent than telomerase activation and has been linked to genomic instability, aggressive tumor behavior, and poor prognosis. Although LTL does not directly reflect telomere dynamics in tumor cells, it has been widely used as a surrogate marker of systemic telomere regulation, reflecting cellular stress and offering insights into disease susceptibility.

In the present study, we evaluated the RTL in peripheral blood lymphocytes to assess telomere dynamics in patients with bone sarcoma and in cancer-free subjects. Our findings revealed that RTL was significantly longer in patients compared to controls. Additionally, stratified analyses by histological subtype showed variable results across groups, with a statistically significant difference observed only in the Ewing sarcoma subgroup compared to controls. The chromosomal instability characteristic of sarcomas may be associated with telomere dysfunction [25, 26]. Consistent with our findings, previous studies have also reported longer telomeres in sarcomas [27, 28]. In a multi-ethnic study, a genetic predisposition for longer LTL was associated with an increased risk of pediatric osteosarcoma [29]. Similarly, Wang et al assessed the TL in pediatric tumors using whole-genome sequencing and reported that patients with osteosarcoma had the most telomere lengthening [30]. Additionally, the authors identified ATRX alterations in 40% of osteosarcoma patients with telomere lengthening.

In the present study, we observed a difference in mean RTL between male patients and male controls, but not between females. Although statistically significant, this finding may reflect differences in sample size and cohort composition. Regarding age, TL is expected to progressively decline over life span. In this study, a trend toward age-related telomere shortening was observed in the control group, albeit not statistically significant, possibly due to limited sample size and age distribution. However, this finding should be interpreted with caution, given the differences in age distribution between patients and controls in both pediatric and adult subgroups, considering the strong age dependency of TL. By contrast, no decline in TL with increasing age was detected among sarcoma patients. Sharaf et al similarly reported higher telomeric content in sarcoma samples from older patients [27]. In contrast, Park et al observed a decrease in RTL with age in sarcoma samples (n = 14) in a cohort of adolescents and young adults with childhood cancer, but no significant difference was found [31].

It has been reported that sarcoma samples genomically complex/lacking specific translocations exhibit elongated telomeres compared to translocation-associated sarcomas [27, 32, 33]. Interestingly, the long TL observed in osteosarcoma samples has been associated with the prevalence of ALT mechanism, whereas a low prevalence of ALT was found in Ewing sarcoma, suggesting predominance of the telomerase-dependent mechanism for telomere maintenance in such cases, possibly driven by the oncogenic fusion *EWS*::*FLI1* [34, 35]. Terasaki et al investigated TL in bone and soft tissue tumors using Southern blotting assay and observed TL heterogeneity, but no significant difference was found [14]. Sharaf et al investigated the telomeric content in 13,555 sarcoma samples using the TelomereHunter tool and identified that the soft tissue osteosarcoma (extraskeletal) had the highest median telomeric content across all sarcoma types [27].

In the present study, no significant association was observed between RTL and outcome events in patients with sarcoma. However, these analyses were limited by the small sample size and a substantial loss to follow-up, resulting in reduced statistical power to draw definitive conclusions regarding the prognostic value of RTL. Sotillo-Pineiro et al investigated pediatric patients with osteosarcoma and Ewing sarcoma and identified that primary tumors had significantly longer TL than metastatic tumor tissues [36]. The authors also detected telomerase activity in 85% of the tumor metastases, whereas only 12% of the primary tumors exhibited telomerase activity. Also, significantly longer event-free survival was observed for patients lacking telomerase activity. Similarly, Ulaner et al demonstrated that patients with osteosarcoma lacking both telomere maintenance mechanisms (telomerase and ALT) had improved survival outcomes [37].

Most studies investigating telomere biology in sarcomas, as discussed above, have focused on TL and maintenance mechanisms within tumor tissue. In contrast, data on LTL in sarcoma patients remain limited. In the present study, longer LTL is interpreted primarily as a potential predisposing systemic factor; however, given the cross-sectional design, a secondary effect of tumor presence or tumor-related systemic responses on leukocyte telomere dynamics cannot be fully excluded. For this purpose, we performed T/S ratio analysis of paired samples in a subset of patients, and our data revealed a consistent intra-individual pattern of TL between peripheral blood and tumor tissue, suggesting that systemic telomere dynamics may be reflected at the tumor level. Additionally, immunohistochemical analysis revealed the heterogeneity of telomere maintenance mechanisms in bone sarcomas. While most cases showed strong TERT expression, ATRX staining patterns were more variable, indicating that distinct pathways may be involved in telomere maintenance within this cohort. Although ALT mechanism is frequently described in sarcomas, TERT expression has also been reported [30, 34, 36]. Together, these findings are consistent with previous studies, supporting that telomerase-dependent and independent mechanisms for telomere maintenance may contribute to bone sarcoma development.

The present study has some limitations, including the relatively small sample size, age group imbalance, and the absence of complementary genetic analyses. Additionally, nine patients (20.5%) were lost to follow-up. An important factor contributing to loss to follow-up is that our institution functions exclusively as an orthopedic referral center, with oncological treatments such as chemotherapy and radiotherapy provided at external institutions. As a result, some patients could not be included in longitudinal outcome analyses.

Previous studies in non-Brazilian populations have also suggested a relationship between LTL and sarcoma susceptibility. A study conducted in a multi-ethnic pediatric and adolescent population reported a significant association between genetically predicted longer LTL and increased osteosarcoma risk, with a stronger effect observed in Hispanic individuals [29]. In this context, our findings extend this evidence by providing, to the best of our knowledge, the first data in Brazilian patients suggesting that dysfunctional telomere maintenance by elongated LTL may be associated with biological mechanisms involved in tumor development, supported by findings in tumor tissue. These findings highlight the need for a deeper understanding of the molecular mechanisms underlying disease in the Brazilian population, which remains underrepresented in genomic research [38]. This is especially crucial for the molecular study of rare cancers, such as sarcomas. Longitudinal studies with larger cohorts will be essential to further elucidate the TL dynamics in patients with sarcoma.

## Data Availability

The data underlying this study’s findings are available from the author upon reasonable request.

## Abbreviations

LTL: leukocyte telomere length
qPCR: quantitative polymerase chain reaction
RTL: relative telomere length
TL: telomere length
